# Optimal Frequency and Wireless Power Budget for Miniature Receivers in Obese People

**DOI:** 10.3390/s23198084

**Published:** 2023-09-26

**Authors:** Tom Van de Steene, Emmeric Tanghe, Luc Martens, Carmine Garripoli, Stefano Stanzione, Wout Joseph

**Affiliations:** 1 Department of Information Technology, Ghent University/imec, B-9052 Ghent, Belgium; 2 Holst Centre/imec, 5656 AE Eindhoven, The Netherlandsstefano.stanzione@imec.nl (S.S.)

**Keywords:** wireless power transfer, miniature implantable medical devices, deep implants, ingestibles, resonant inductive power transfer, numerical simulations, coil design

## Abstract

This study investigates wireless power transfer for deep in-body receivers, determining the optimal frequency, power budget, and design for the transmitter and receiver. In particular, the focus is on small, in-body receivers at large depths up to 20 cm for obese patients. This enables long-term monitoring of the gastrointestinal tract for all body types. Numerical simulations are used to investigate power transfer and losses as a function of frequency and to find the optimal design at the selected frequency for an obese body model. From all ISM-frequencies in the investigated range (1 kHz–10 GHz), the value of 13.56 MHz yields the best performance. This optimum corresponds to the transition from dominant copper losses in conductors to dominant losses in conductive tissue. At this frequency, a transmitting and receiving coil are designed consisting of 12 and 23 windings, respectively. With a power transfer efficiency of $2.70\times 10^{-5}$, 18 µW can be received for an input power of 0.68 W while still satisfying exposure guidelines. The power transfer is validated by measurements. For the first time, efficiency values and the power budget are reported for WPT through 20 cm of tissue to mm sized receivers. Compared to WPT at higher frequencies, as commonly used for small receivers, the proposed system is more suitable for WPT to large depths in-body and comes with the advantage that no focusing is required, which can accommodate multiple receivers and uncertainty about receiver location more easily. The received power allows long-term sensing in the gastrointestinal tract by, e.g., temperature, pressure, and pH sensors, motility sensing, or even gastric stimulation.

## 1. Introduction and Objectives

### 1.1. Long-Term Monitoring with Implants and Ingestibles

For more than a century, investigations of the gastrointestinal tract (GI tract), or organs in the abdominal cavity in general, have become more and more accessible with the rapid evolution of endoscopic techniques [1]. A trend toward increasingly less invasive methods can be noticed, starting with the more flexible fiber-optic endoscopes [2], to even swallowable devices for Wireless Capsule Endoscopy (WCE) [3]. These evolutions significantly increased the comfort of the patient and allowed for the examination of the complete GI tract. As a result of normal, peristaltic movements, mean total transit times for WCE are around 24 h, with a small bowel transit time varying from 45–240 min [3,4]. This eliminates the possibility of long-term monitoring. However, pH, temperature, and pressure measurements in the GI tract can provide valuable insight into (patho-)physiology, e.g., in the case of delayed gastric emptying [5].

Similarly to wearables, which have been measuring vital signs and tracking sleep for years now, ingestible, implantable, or injectible devices could be of high value, linking parameter sensing in the GI tract to health and disease [6,7]. Temperature measurements by in-body devices have the highest accuracy, least delay, and most comfort for the patient. They can be used to detect GI bleeding [8], menstrual disorders [9], polycystic-ovary syndrome [10], depression [11], or infection [12]. Measurements of pH value in the stomach can be used to diagnose gastroesophageal reflux disease [13]. Pressure sensors can detect intra-abdominal hypertension [14]. Devices for microbiome and biomolecule sensing in the GI tract allow us to elucidate their yet largely unknown relation with the etiology of many diseases, including Crohn’s disease, ulcerative colitis (a chronic inflammatory bowel disease), or Menetrier disease [7,15,16,17,18]. Sensing biomarkers, such as thiosulfate and acyl-homoserine lactone, could indicate inflammation or infection of the gut [15]. The opportunities for long-term monitoring in the abdominal cavity are not limited to inside the GI-tract: electrophysiological signal measurements with a miniature sensor from [19] in the adrenal gland were correlated with elevated cortisol and chronic stress. Implants for functional electrical stimulation along the GI tract exist as well, e.g., [20]. Gastric stimulation can improve health for morbidly obese patients, intestinal stimulation is used for post-operative ileus management, and colon stimulation can control the movement of solid content in the bowel [20,21]. In order to keep the device at a certain location in the body, a retention mechanism is needed. Implants such as in [20] can be fixed, but need a more invasive procedure. A tissue attachment mechanism (TAM) was developed by [22], which allows a sensor to clip on the inner intestine wall. This is done using a capsule robot with its only task being to install the sensor at a specific location. Autocapsule [23] is a similar example of a device meant to stay in the GI-tract for prolonged monitoring. Ghosh et al. [24] developed a multi-clawed gripper, inspired by the hookworm parasite, that can latch onto the mucosal tissue for prolonged stay in the colon. The intervention is conducted via the anus in a minimally invasive way.

### 1.2. Powering Miniature Medical Devices

One of the biggest challenges for these miniature devices is providing power for long monitoring durations [25]. In particular, for obese people, for who the separation between transmitter (Tx) and receiver (Rx) can be up to 20 cm, providing power to the hardware can be difficult.

Batteries are suitable for applications such as WCE, since regular silver-oxide button cells fit in a typical capsule, having dimensions of 32 mm × 11 mm diameter [26,27]. Nonetheless, batteries come with some serious disadvantages. They can be toxic, have a finite capacity, limited shelf life, poor disposability, and could cause mucosal injury [28,29,30,31]. But most importantly, the specific energy density of batteries decreases drastically for small form factors: a 16 mm${}^{3}$ battery comes with an energy density of only 26 mWh/cm${}^{3}$ [6], which makes batteries unsuitable for long-term use in miniature devices.

Supercapacitors have even lower energy densities at around 2.5 mWh/cm${}^{3}$ [32].

Ultrasound (US) power transfer, on the other hand, could be a viable option for implanted and ingestible medical devices. Due to the lower speed of US waves, the wavelength becomes smaller at lower frequencies already, compared to electromagnetic waves, and also losses are lower, enabling a relatively efficient power transfer, particularly for small receivers [29,32]. From a practical point of view, the need for gel to provide good contact between transmitter and body tissue is rather inconvenient. The concept of focusing also implies that only a small spot inside the body will be ideal for maximum power transfer, and tighter tolerances are applicable for the placement of the transmitter. This makes US power transfer less desirable for deep implants in obese people, where the relative position of transmitter and receiver can already be influenced by the posture of the patient. Lastly, power transfer efficiencies reported in the literature can be an overestimation since often only a single tissue type is considered, while the interfaces between different tissue types might induce inefficiencies during power transfer [32,33].

Capacitive coupling power transfer is also known to be inapplicable for large separation and small device size [25,34,35].

Energy harvesting from the surroundings is a third option, next to local storage and wireless power transfer. Unfortunately, the power harvesting mechanism might be specific to a certain anatomical location, or power values could become too low [36]. For example, energy is successfully harvested by a miniature receiver in the stomach in [31], but the received power drops significantly to the order of a few nW/mm${}^{2}$ once the receiver enters the intestine. Power can be harvested from body movements as well [37]. This obviously means that power is received only when the body is in motion, which is not always the case, both for the general population and for obese people [38].

Considering the shortcomings of the mechanisms discussed above, this study will investigate electromagnetic wireless power transfer for deep, miniature medical devices, which is arguably the most common type of WPT.

### 1.3. Novelty and Objective

**Figure 1:**
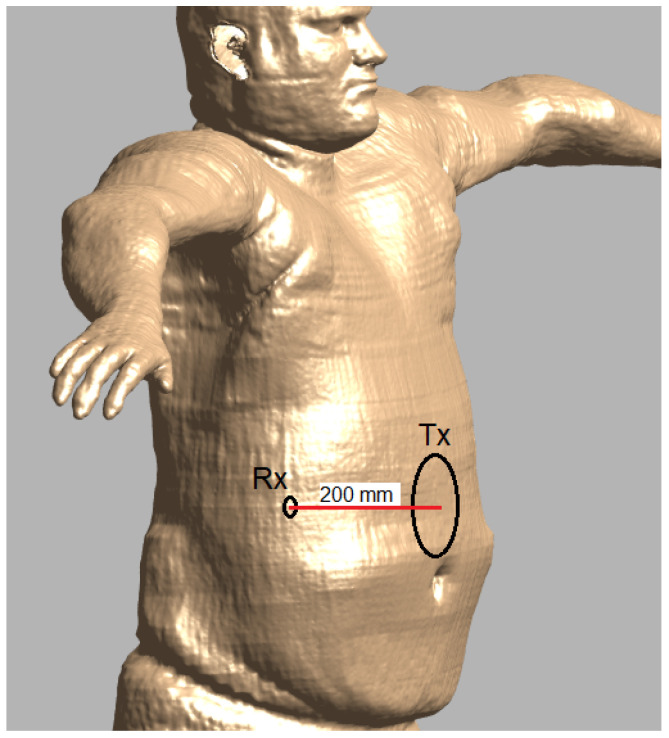
Application goal: Wireless power transfer to in-body receivers for obese people. Adapted from [53].

This study aims to determine an optimal frequency and design for wireless power transfer to a small, in-body receiver for obese people by means of numerical simulations, aiming for maximum power transfer efficiency for the given application. The general concept is illustrated in Figure 1. We explicitly aim for a power transfer distance of 200mm. This distance can cover the whole body for an abdominal circumference of up to 130 cm, corresponding to BMI-values of over 40 [39]. A maximum radius of 2 mm by a thickness of 2 mm is imposed as the maximum dimensions of the receiver. This allows the total device to be small as well and stay in the body for prolonged durations. The dimensions of the transmitter are constrained to 100mm for the diameter and 10mm thickness. An overview of the geometric constraints is shown in Table 1. While the main objective is maximum power transfer efficiency, also the received power is important with regard to the range of possible applications. The maximum received power is a result of the limitations on input power at the transmitter, for reasons of safety in terms of exposure of the body to electromagnetic fields. The first step will consist of determining the optimal frequency, without excluding near-field, mid-field, or far-field beforehand. This will be based on mapping the main loss mechanisms during power transfer and, most importantly, how they change with frequency. In a second step, the design for the transmitter and receiver will be defined, with the goal of optimal power transfer efficiency.

The novelty of the paper lies in the following three main points:

Ultra-deep, miniature receivers: For the first time, mm sized receivers are investigated at depths up to 20 cm in the body, for the inclusion of all body types. Existing studies either focus on smaller depths, <10 cm, for deep implants [36,40,41,42,43,44], target larger receivers [45,46,47], or include tissue only for a small part of the Tx-Rx distance [48].
Frequency methods and value: The approach of starting from the complete electromagnetic spectrum, from near-field inductive methods to far-field radiation and identifying the main losses at each frequency, allows us to find a maximum efficiency point. For the first time, this study explicitly focuses on the optimal efficiency for a depth of 20 cm. Interesting studies searching for the optimal frequency have been conducted already [40,49,50,51,52], although tissues are considered homogeneous, a far-field approximation is used, only tissue losses are considered for the efficiency, or the depth is limited to 10 cm while the optimal frequency for WPT significantly depends on depth and tissue characteristics.
  Secondly, the frequency of 13.56 MHz that will ultimately be used for the Tx and Rx design, has benefits compared to the more common mid-field and far-field approaches, as no focusing with the feedback loop is needed, the fields and power transfer are almost insensitive to variable tissue properties, and losses in electronics will we lower. The large wavelength gives this approach the benefit of transferring power to multiple receivers in the abdominal cavity more easily, undoubtedly a benefit for the application of long-term monitoring.
User comfort: The limitations on Tx dimensions ensure that the hardware has a convenient form factor: A 10 cm coil can easily be worn on the body without hindering. A coil worn around the body as in [45,46] would be more cumbersome, needs an electrical connection to close the loop, and would require individual sizing. No need for contact gel as for US WPT also improves the user experience.

## 2. Models and Methods

The study consists of two main steps. In the first step, the optimal frequency for the use case will be determined, considering the intended distance for power transfer and typical tissue characteristics. Using this frequency, the optimal transmitter and receiver will be designed in a second step, taking into account the geometric constraints of the system.

### 2.1. Simulation Software and Models

The simulations are run using the COMSOL Multiphysics v5.6 simulation software [54]. Both the low-frequency (ACDC) and high-frequency (RF) modules are used. This software allows simulating both 2D- and 3D-domains, at virtually all frequencies. The material properties are taken into account as a function of frequency as well, as opposed to the more limited magnetic simulators.

**Figure 2:**
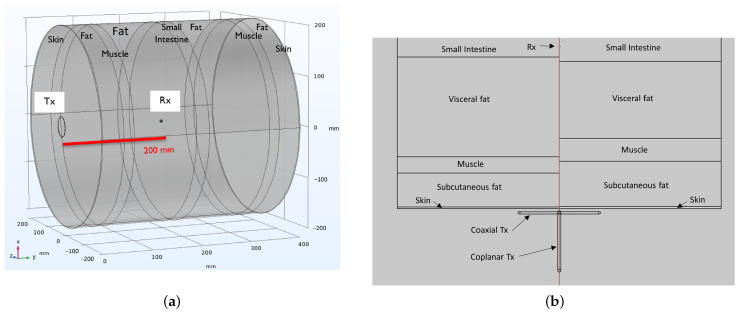
The tissue model, consisting of symmetrically stacked cylindrical layers of tissue. (**a**) shows the model in 3D. The transmitter, consisting of a single loop coil is shown at the left base of the cylinder. The receiver is located inside the model, although very small. (**b**) shows a cross-section of the model. the left side shows (half of) the original model, the right side shows (half of) the model with modified tissue thickness. Both the coaxial and coplanar configurations are shown for Tx. Rx is very small, and is parallel to Tx.

The power transfer was simulated using 3D numerical models of the transmitter, receiver, and tissue layers in between. This model is shown in Figure 2a,b. The thickness of the tissue layers was such that they sum up to 200mm, and electrical properties from Gabriel et al. were used [55]. The thickness of each layer is listed in Table 2. The layers were cylindrical (Figure 2a), with the axis along the line Tx–Rx, and the diameter of 400mm was chosen to be large compared to the Tx–Rx distance. A second model with modified layer thickness was composed as well. This enables us to assess the influence of the body model and variations of the electrical properties of the body on the power transfer. In this modified model, also listed in Table 2, the amount of visceral fat is reduced, and the thickness of other layers is increased, taking into account the fixed total depth of 200mm. These changes are random and will be used to compare the influence of tissue properties between frequencies in a relative manner. At low frequencies, the cylindrical symmetry allows much faster simulations for finer parameter steps and for validation of the 3D-model.

### 2.2. PTE in a Two-Port Network

**Figure 3:**
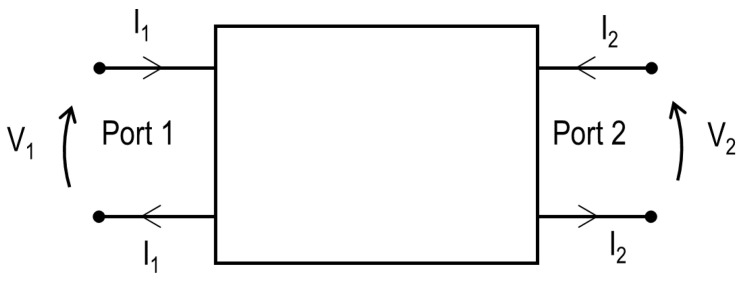
A general two-port network. The relation between voltages and currents at each of the ports is given by the matrix of Z-parameters in Equation (1).

The power transfer efficiency (PTE) is quantified by considering a general, reciprocal two-port network between the transmitter and the receiver, as illustrated in Figure 3 and Equation (1). The efficiency (PTE) can be expressed as a function of the Z-parameters, Equation (2), and is defined in this study as the ratio of the maximum power consumed by a matched load to the input active power provided by the source.

(1) $$\left[\begin{array}{c} V_{1}\\ V_{2} \end{array}\right]=\left[\begin{array}{cc} Z_{11} & Z_{12}\\ Z_{21} & Z_{22} \end{array}\right]\left[\begin{array}{c} I_{1}\\ I_{2} \end{array}\right]$$

(2) $$PTE=\frac{|Z_{21}{|}^{2}}{4R_{1}R_{2}}$$

R1=Re(Z11),R2=Re(Z22)

Equation (2), derived from [56], is valid when the coupling is low, i.e., when the mutual impedance is much lower than the real parts of both input impedances, and is, therefore, valid for low values of the PTE. Due to the large separation of 200mm between Tx and Rx, this approximation is justified. This equation clearly shows the influence of each of the Z-parameters. For the input impedances, $Z_{11}$ and $Z_{22}$, only the real part is of importance. This corresponds to the fact that the reactive power has no influence on the PTE, being the ratio of active powers. For the mutual impedance $Z_{21}$, only the magnitude should be considered. The expression for PTE can be derived from the ratio of output to input power as well.

For the case of low coupling, the voltage and current at the receiver port will be very small compared to the transmitter port. As a result of this very low output power, the input power equals, in good approximation, the sum of all the losses and is represented by $\frac{1}{2}{\left|I_{1}\right|}^{2}\cdot R_{1}$. Low coupling also allows us to consider the receiver port as a voltage source with voltage $I_{1}\cdot Z_{21}$ and internal resistance $Z_{22}$. The maximum power that can be drawn, considering a matched load $Z_{load}=Z_{22}^{*}$, equals $\frac{1}{8}\frac{|I_{1}{|}^{2}\cdot {\left|Z_{21}\right|}^{2}}{R_{2}}$. The ratio of the powers corresponds to Equation (2).

### 2.3. Optimal Frequency

The first step of this study consists of finding the optimal frequency for this use case. To compare values of the achievable efficiency at a wide range of frequencies, a model with a single-loop coil for Tx and Rx is used. The diameter of the Tx coil is set to 10cm, which represents a reasonable size for a device to be worn on the body. The Rx coil diameter is fixed at 3.3mm.

All parameters in Equation (2) can be determined using 2 simulations for each frequency point. A first simulation with a current of 1A in Tx and open Rx will provide $R_{1}$ and $Z_{21}$. A second simulation with 1A in Rx and open Tx provides $R_{2}$, and can act as a check for $Z_{21}$, which is equal to $Z_{12}$.

The first simulation suffices to investigate the different loss mechanisms: for a fixed input current, $R_{1}$ represents the total input power. Due to the small coupling, a small Rx current is expected, and corresponding losses will be small as well. However, the role of $R_{2}$ is equally important, illustrated by the symmetry of Equation (2), representing the internal resistance of the magnetically induced voltage source at Rx. Since the load should be matched to this impedance, the received power is inversely proportional to its real part.

#### 2.3.1. Maximum Efficiency

Multiple causes of losses can be distinguished. The frequency sweep serves to investigate these different mechanisms and, more importantly, their change with frequency. The most important loss mechanisms include copper losses in the transmitter and receiver and tissue losses due to induced currents and dielectric relaxation.

The range of investigated frequencies varies from 1kHz up to 10GHz. At low frequencies, the PTE is expected to increase with the square of the frequency since the induced voltage in Rx is proportional to the frequency of the magnetic field. The rate of increase will be attenuated by an increased resistance of the Tx coil as a result of the skin effect. At even higher frequencies, tissue losses will become increasingly important. Due to the non-zero conductivity of biological tissue, the associated losses caused by induced currents will limit the power transfer efficiency proportionally to the square of the frequency [57]. The power lost due to each of these loss mechanisms can be identified during the simulations, providing insight into the trend for the final PTE as a function of frequency.

#### 2.3.2. Robustness

The PTE and corresponding optimal frequency are highly dependent on the design of the transmitter and receiver, including type, size, and orientation, as well as the electrical properties and dimensions of the tissue layers. A model with modified tissue layer thickness, as explained above, is used to assess the influence of tissues with varying conductivity and permittivity. This is shown in Figure 2b. Additionally, multiple orientations of the Tx- and Rx-antenna are simulated. This accounts for the fact that the radiation pattern of a loop varies with frequency: a small loop will radiate with maxima in the plane of the loop, while a loop near resonance will radiate maximally along its axis. This is shown in Figure 2b as well.

### 2.4. Transmitter and Receiver Design

In the second step of this study, a coil is designed for the Tx- and Rx-side for WPT at a frequency of 13.56MHz, as found in Section 3.1. At this frequency, the wavelength in tissue is in the order of meters, depending on the exact tissue type. The largest wavelength is found in fat, which covers the largest part of the 20cm separation between Tx and Rx, with a value of 6.4m. In the small intestine, the value for the wavelength is the smallest at 1.2m, but the distance that the field has to travel through the small intestine is only a matter of millimeters to centimeters as illustrated in Figure 2a. Since the typical dimensions of the simulation domain are considerably smaller than the wavelength, the low frequency solver is used to optimize the Tx and Rx coil. The AC/DC module of the COMSOL Multiphysics v5.6 software [54] is used for this step in combination with the 2D axisymmetric solver.

#### 2.4.1. Optimization Methodology

The designs of the Tx and Rx coils are defined by 5 independent parameters, in addition to the fixed outer dimensions of both coils.

- $N_{a}$, the number of windings in the axial direction;
- $N_{r}$, the number of windings in the radial direction;
- *d*, the winding diameter;
- $i_{a}$, the winding spacing in the axial direction;
- $i_{r}$, the winding spacing in the radial direction.

**Figure 4:**
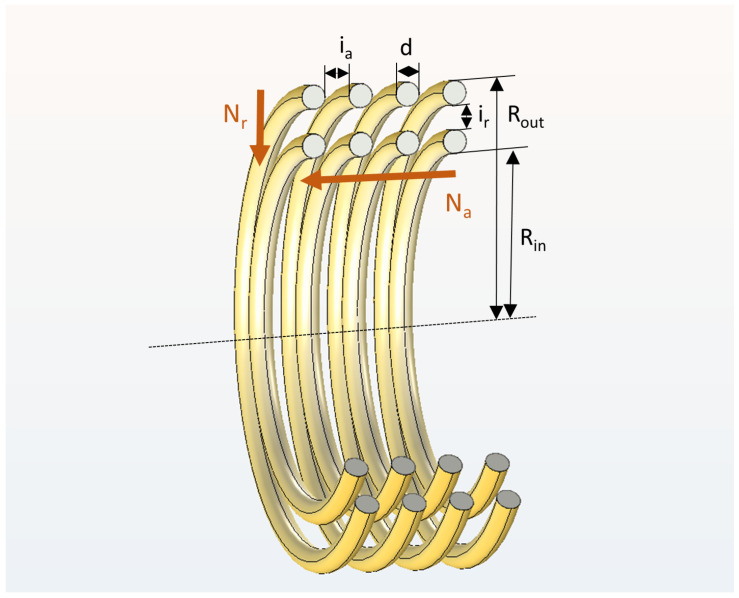
Cross-sectional view of the Tx coil, illustrating the definition of each of the investigated parameters: axial and radial number of windings ($N_{a}$ and $N_{r}$), outer and inner radii ($R_{out}$, $R_{in}$), winding diameter (*d*) and winding spacing ($i_{a}$ and $i_{r}$).

The role of these parameters is also illustrated in Figure 4. The optimization consists of a grid-search algorithm. This can be used due to the very fast simulation time of the axisymmetric solver. If the time allows for a grid-search algorithm, it will always be the most complete optimization algorithm. Furthermore, the parameter spaces are well confined as well: $N_{a}$ and $N_{r}$ are limited to positive integers of modest magnitude, after which only 3 continuous parameters remain. The ranges for all parameters are shown in Table 3. After a first iteration, the ranges for each parameter could already be limited and refined, allowing for an accurate identification of the optimum.

Nonetheless, it turns out that the definition of this optimum is less straightforward. Although PTE is the main objective of the optimization, it is important to consider the coil resistance as well since it will influence the performance and efficiency of the system as a whole. Since losses in circuits are generally proportional to the square of the current, transmitting and receiving power at a high voltage/current ratio improves overall efficiency. In particular, at the receiving side, where power and voltage values are already low, a sufficiently high voltage is needed. The load circuits performing AC-DC rectification are necessarily non-linear in nature. This implies that they will have different efficiencies for different voltage amplitudes. Typically, they contain solid-state devices with a certain threshold voltage, meaning that under a certain voltage, the total energy efficiency will collapse. Despite the fact that a parallel capacitor will also significantly increase the voltage of the resonating circuit, a larger current in the resonating circuit will imply larger losses due to the capacitor equivalent series resistance. Additionally, a small resistance value produces a small resonance peak in the frequency domain, requiring small tolerances on the electric components to tune the coil, which, in turn, increases the sensitivity to objects in the environment of the coil, including the body. A value for $R_{1}$ and $R_{2}$ above 1Ω will ensure good compatibility and efficiency of the source and load circuits.

#### 2.4.2. Transmitter Optimization

Due to the low coupling, the optimization of Tx and Rx can be done separately. For the optimization of Tx, a simple single winding is used to model the Rx coil to limit the complexity of the simulation. The Tx coil will be fed with a current of 1A, while the Rx coil is left open, i.e., a current of 0A is imposed. The voltages in both coils and resistance of Tx are directly obtained from the simulation results. $Z_{21}$, which is of importance to calculate the efficiency, is obtained from the open Rx coil voltage. To compare the efficiency of multiple Tx designs, the expression for the PTE (2) is employed with a fixed design for Rx, consisting of a small, single loop. The corresponding value for $R_{2}$ is 5.7 mΩ.

While the maximum outer dimensions are already fixed at 50mm and 10mm for the radius and thickness of the coil, respectively, the five independent parameters in Table 3 remain for the optimization of the coil. The parameter grid is simulated as explained in Section 2.4.1 and the optimal design of the coil will be selected, based on a trade-off between efficiency and resistance, which is discussed in the results.

#### 2.4.3. Receiver Optimization

The approach used for the optimization of the Rx is analogous. However, the dynamic range of the dimensions in the simulations would be very large if we would model the complete domain: the largest dimensions of the model with Tx and Rx (multiple decimeters) are much larger than the geometric features of the Rx coil (multiple windings per mm). Therefore, only the Rx coil and its environment are simulated. The magnetic field at the location of the receiver is assumed to be constant, due to the large distance (200mm) from the transmitting coil and the small dimensions of the receiving coil (4mm). This significantly simplifies and speeds up the simulations. The best performing coil will again be determined by a combination of high efficiency and sufficiently large input impedance.

## 3. Results and Discussion

### 3.1. Optimal Frequency

#### 3.1.1. Efficiency Values

Results are shown for the coaxial configuration of the coils first. Subsequently, the robustness of the model is investigated by changing a number of model parameters, as described in Section 2.3.2.

**Figure 5:**
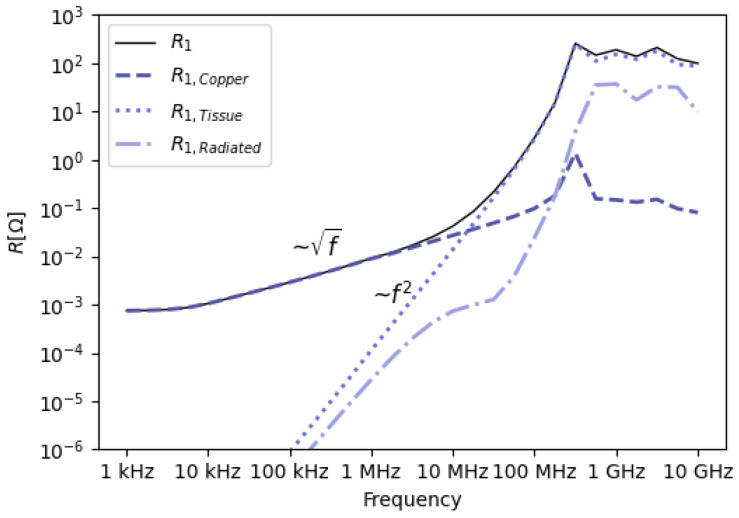
Contributions to $R_{1}$, showing the influence of copper losses ($R_{1,Copper}$), tissue losses ($R_{1,Tissue}$), radiation losses ($R_{1,Radiated}$) and the total sum ($R_{1}$) for a fixed input current $I_{1}$ = 1 A.

The composition of $R_{1}$ reveals valuable insight into the origin of the losses present at each frequency point. This is shown in Figure 5. As a result of the fixed input current of 1A, the resistance $R_{1}$ can be directly interpreted as the input power.

At low frequencies, the only loss component of $R_{1}$ is the copper loss of the transmitter, which is constant at sufficiently low frequencies. At around 1kHz, the skin depth becomes considerably smaller than the diameter of the Tx winding. Since skin depth is proportional to the square root of the frequency, the Tx resistance, therefore also the input power, increases with the square root of the frequency from that point on, as indicated in Figure 5.

Starting at MHz frequencies, induced currents and therefore absorbed power in conductive tissue becomes progressively important. The imaginary part of the permittivity due to dielectric relaxation of the tissue, induces increasingly larger losses at higher frequencies. From 20MHz on, this becomes the dominant loss mechanism, as a result of the relation with the square of the frequency. Copper losses in Tx and received power by Rx become very small relative to the power absorbed in tissue, which is nearly equal to the input power.

Starting at approximately 100MHz, the radiated power constitutes a significant portion of the input power. It is important to note that only power radiated out of the simulation domain is considered radiated power. Power radiated by the Tx coil and absorbed by the tissue is counted as tissue loss.

At lower frequencies, it makes sense for the visualization to fix the input current of the loop: the input current is equal to the current in the loop, and the real part of the input impedance is directly related to the loss components. When the wavelength becomes smaller, the current in the loop cannot be considered constant anymore, and the input impedance of the loop can become either very high or very low, depending on the frequency. Consequently, a change in the components of $R_{1}$ can either be attributed to a change in the respective loss mechanisms, or to a change in input impedance. The latter implies that at these higher frequencies, the loss components should be interpreted in a relative manner and explains the irregular shape of the graphs above 100 MHz in Figure 5.

**Figure 6:**
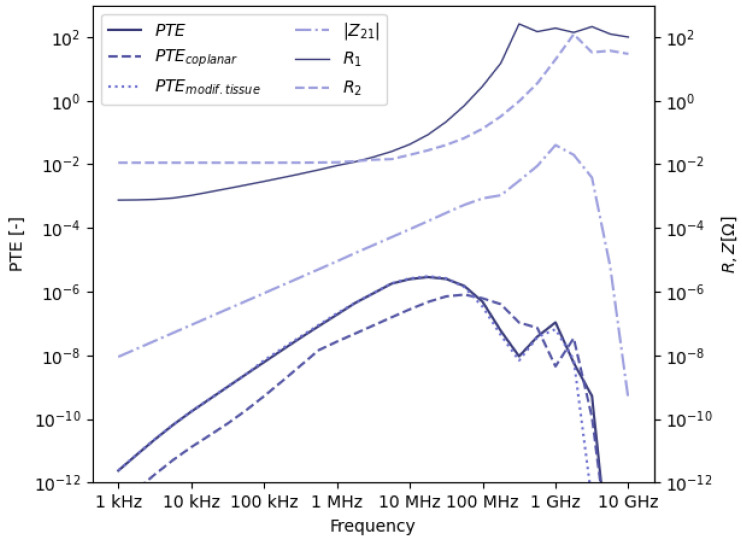
Efficiency of the single-loop model at multiple frequency points. The difference between the original model (PTE) and the model with modified tissue layers ($PTE_{modif.tissue}$) is small and the graphs overlap at frequencies up to 100MHz. The coplanar model ($PTE_{coplanar}$) mainly differs at lower frequencies, where the latter yields lower efficiency. The influence of all factors contributing to the PTE (Equation (2)) is shown as well ($|Z_{21}|$, $R_{1}$, $R_{2}$).

Figure 6 shows the total PTE with its factors according to Equation (2): $|Z_{21}|$, $R_{1}$ and $R_{2}$. The progress of $R_{2}$ can be explained similarly as for $R_{1}$, although the transition from dominant copper losses to dominant tissue losses occurs at a higher frequency, due to the smaller dimensions and winding diameter.

At the receiving side, the rate of change of the magnetic field and therefore the induced voltage, represented by $|Z_{21}|$, is initially proportional to the frequency, because the magnetic field is proportional to the (constant) current in the transmitter. At higher frequencies, the inductive link transitions to radiative power transfer, ceasing the linear relationship between induced voltage and frequency, as indicated in Figure 6. In particular, at higher frequencies, tissue losses will reduce the available power at Rx.

#### 3.1.2. Choice of Optimal Frequency Value

The graph of the PTE in Figure 6 reveals a maximum at 17MHz. At this point, the denominator in Equation (2) increases faster with frequency than the numerator. This value results from the transition from dominant copper losses to dominant tissue losses. This means there are some implications when deciding on the ideal frequency for WPT to receivers located deep in the body. Since the transmitter and receiver are not yet optimized for power transfer at a specific frequency, the indicated PTE will be an underestimation of the maximally achievable efficiency. Nevertheless, lower copper losses as a result of optimized Tx and Rx design will mainly increase the PTE at lower frequencies. At frequencies where tissue losses are dominant, the expected increase will be lower. In the latter case, a more directive electromagnetic field could result in reduced tissue losses and increased power transfer. However, this would significantly complicate the design of the complete system, since the exact location of the receiver with respect to the transmitter should be known and the shape of the field should be adapted accordingly. Moreover, the wavelength should be considerably smaller than the value of approximately 2m at 17MHz (in tissue). Consequently, lowering the tissue losses at higher frequencies will be hard to achieve. A last consideration to take into account is the availability of the frequency for the intended application. Since ISM-frequencies are free to use, while having bands along the complete spectrum, they are of particular interest. The ISM-frequency closest to the maximum at 17MHz is 13.56MHz. This value is selected for the further optimization of the WPT system, considering the limited difference in PTE and the relatively larger potential for the optimization of copper losses in the transmitter and receiver. Considering the large frequency spectrum, this value is in good agreement with the value of 4 MHz found in [46].

#### 3.1.3. Robustness

The choice of optimal frequency should be independent of the Tx and Rx models that are used since they do not necessarily represent the most efficient design. Similarly for the used body model, variations in anatomy will be present between different patients, even if the total distance is the same. Therefore, the dimensions of each tissue layer are modified according to Table 2. As illustrated by the dotted line labeled $PTE_{modif.tissue}$ in Figure 6, this had negligible influence on the PTE curve and maximum value. Another advantage at 13.56MHz is that the shape of the magnetic field is not changed significantly due to the heterogeneous body, as opposed to the very irregular behavior of electromagnetic waves in the body at microwave frequencies. This limits high local variations of the PTE. Furthermore, the polarization of the loop antenna was changed to a coplanar configuration (dashed line $PTE_{coplanar}$ in Figure 6), where both Tx and Rx loops are in the same plane, perpendicular to the skin. At lower frequencies, below 100MHz, power transfer is less efficient compared to the default, coaxial configuration. At higher frequencies, the efficiency shows more fluctuations in the PTE curve, as does the coaxial model, but the order of magnitude of both configurations is similar. In particular, for receivers in the GI tract, small shifts in position due to external movement or internal organ movement can cause these fluctuations in efficiency, which are unwanted. As a result, the value of 13.56MHz is used in this study.

### 3.2. Transmitter Optimization

**Figure 7:**
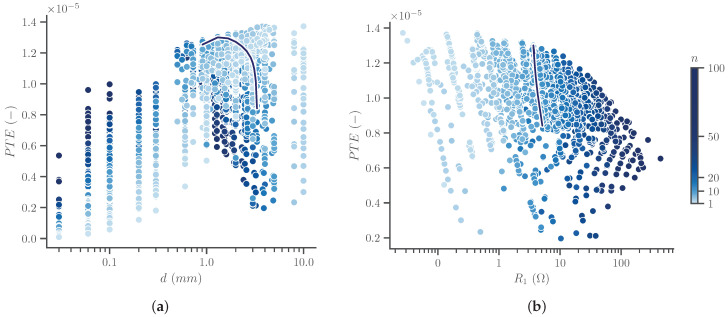
Simulated PTE for multiple Tx designs as a function of (**a**) winding diameter and (**b**) input resistance $R_{1}$. The color shows the total number of windings, $n_{a}\cdot n_{r}$. A supplementary blue line is drawn, showing the trend for all points with the final number of 12 windings.

Using the optimal frequency of 13.56MHz, simulations were carried out to obtain the optimal design for the transmitter using the parameter ranges in Table 3. The efficiency, according to Equation (2), is shown as a function of the winding diameter in Figure 7a, with colors indicating the total number of windings.

Figure 7a already shows the expected efficiency range, which is in the order of magnitude of $10^{-5}$, although this is for a sub-optimal Rx. Higher efficiency values are seen for relatively large winding diameter values, which of course corresponds to a limited total number of windings *n*, considering the maximum dimensions of Tx. All cases with a diameter of 1mm or larger are retained for further selection. PTE is similar at these values, while it drops when the diameter is too small. The color scale clearly shows that a higher number of windings is more efficient for low diameter values, while the opposite holds for larger diameters. The dark blue line in Figure 7a shows the trend for a fixed number of 12 windings, corresponding to the final design. This clearly shows that the maximum is reached for an intermediate value of winding diameter, between the minimum and maximum. This is confirmed in Figure 7b: a larger diameter for a fixed number of 12 windings slightly increases resistance and therefore reduces efficiency.

Figure 7b shows the obtained subset of simulations as a function of input resistance $R_{1}$. A minimum requirement on this quantity of 1Ω is imposed. Although the highest efficiency value according to the definition of the PTE is excluded as a result of this requirement, this is not a problem as the total efficiency, including the matching network, would not be maximal for that case.

A final selection from the designs in Figure 7b is made, containing only the coils with the highest efficiency, compared to all cases that have a higher or equal input resistance $R_{1}$. This allows us to make a trade-off between efficiency and input resistance. The specifications of these 12 models are shown in Table 4, sorted by descending efficiency.

While the first option in Table 4 has the highest PTE, the third option has a much higher input resistance. Due to the very small difference in efficiency, the third option is the best choice. The input resistance of 3.7Ω is also sufficient, so the drop in efficiency of the remaining options is not gainful.

The proposed design with 3 by 4 windings in the axial and radial directions, respectively, performs optimally for this specific use case. The PTE after Tx optimization amounts to $1.3\cdot 10^{-5}$. Considering the very small size of the receiver, the large depth of 20cm inside an obese person and the remaining potential of optimization of Rx, this is a satisfying result.

### 3.3. Receiver Optimization

A similar approach that was used for the transmitter is used for the receiver, as discussed in Section 2.4.3. The biggest limitation on the efficiency will be the small size of the receiver. For this application, this size is limited to a diameter of 4mm and a thickness of 2mm. The variables left to determine are the number of windings in the axial and radial direction, the winding diameter and the spacing in both directions.

**Figure 8:**
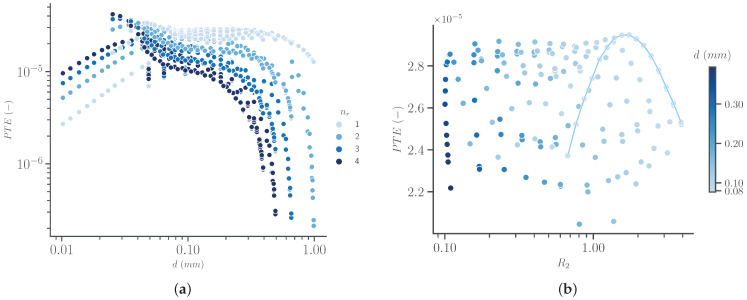
Efficiency of Rx (PTE, vertical axis) for multiple designs, showing the relation with (**a**) winding diameter (d, horizontal axis), radial number of windings (color) and (**b**) the resistance $R_{2}$ (horizontal axis) and winding diameter (d, color) for a fixed radial number of windings of 1. The light blue line shows all points with a winding diameter of 0.079 mm.

Figure 8a shows that a small number of windings in the radial directions is preferred when the winding diameter is not too small. From a practical point of view, this is beneficial, since more space inside the coil is still available for other hardware, as long as it does not considerably influence the magnetic field. At very small winding diameters, i.e., smaller than 0.04mm, this no longer holds, but the efficiency for these cases is lower, while these extremely small diameter values might be associated with a more complex manufacturing process.

If we take into account standard copper wire sizes, the smallest conventional size, as defined by the American Wire Gauge (AWG) [58], is AWG 40, corresponding to a diameter of 0.079mm. Limiting the possible designs to the cases with this minimum diameter, only slightly affects the maximum efficiency, with the benefit of a more conventional design.

Confining the parameter space to all cases with a single winding in the axial direction results in only two remaining degrees of freedom: the axial number of windings and the winding diameter. The axial spacing will follow after choosing these two values. A finer step of the remaining parameters is used for further optimization. The diameters are set by the AWG-value, which are logarithmically spaced with 20 values per decade, starting at 0.079mm, as discussed above. The number of axial windings for each diameter is determined by the whole numbers between 1 and the maximum possible number considering the thickness of 2mm. The simulation results are shown in Figure 8b.

Combining the results from Figure 8a,b shows that the winding diameter has a relatively small effect on the PTE, with slightly higher PTE values for smaller diameter values. However, for a smaller diameter, there will also be a higher number of windings, with a smaller cross-section, resulting in a much higher resistance. This is beneficial with regard to the matching and load circuit. The light blue line in Figure 8b, connecting all points with a diameter of 0.079mm, clearly shows that for a fixed diameter, either too many windings (tight spacing) or too few windings (large spacing) negatively affects efficiency.

The maximum efficiency value in Figure 8b corresponds to a design with 18 axial windings with the smallest winding diameter of 0.079mm. This design has a resistance of 1.7Ω and an efficiency of $2.95\times 10^{-5}$. Depending on the load-circuit, a design with 23 windings might provide a better resistance of 3.1Ω at the cost of a slightly lower efficiency of $2.70\times 10^{-5}$. However, the advantage of the latter is that the power is delivered at a higher voltage and lower current, limiting losses in components and conductors of the load circuit. For practical reasons, the design with 23 windings might be preferred as well since the winding spacing of 0.01mm is a better match with the thickness of the insulation of typical, commercially available AWG 40 wire. This eliminates the need for accurately dimensioning the spacing between two windings. The coil can instead be wound with windings one next to the other.

### 3.4. Exposure and Output Power

Magnetic, electric and electromagnetic fields allow us to communicate and send power to devices inside the body. Unfortunately, these fields are partly absorbed by the body and can be harmful as well when they are too strong. Hence, in this section, this effect is quantified and compared with the applicable guidelines.

#### 3.4.1. Simulations of Exposure

Limitations on electric, magnetic or electromagnetic fields in the body for safety reasons will limit the maximum amount of power that can be sent into the body. These restrictions are published by both ICNIRP [59] and IEEE [60]. At a frequency of 13.56MHz, the limiting quantity is the specific absorption rate, or SAR. This is the rate at which energy from an electric/magnetic field is absorbed by conductive tissue in the body. The limits of both ICNIRP and IEEE are 2W/kg for local exposure of the head and torso, averaged over 6 min and 10g of tissue. ICNIRP does not impose limits on the peak SAR within this 6min window at 13.56MHz. IEEE limits the average SAR for pulsed or non-constant exposure to a fifth of the value for a continuous wave, or 0.4W/kg averaged over every 100ms instead of 6min.

**Figure 9:**
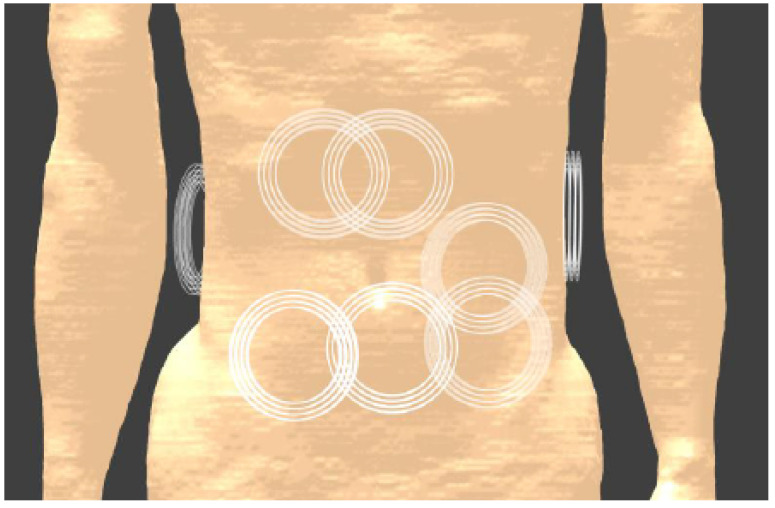
Simulation model to investigate exposure during WPT. The Tx coil (represented by the white circles) is placed at 10 different, randomly distributed locations around the abdomen. During every simulation, only one Tx coil is present.

Exposure of the body to the fields from the coils can be simulated with the Sim4Life v6.2 software [61], using a model of the coil and the adult Duke body model from the Virtual Population [53], as shown in Figure 9. In the final configuration, multiple transmitters will be worn as a belt around the abdomen. To account for variations in the exact placement of the belt and, therefore, the exact location of the transmitting coil, multiple locations are tested, as illustrated in Figure 9. A total of 10 possible locations of the transmitting coil is simulated and the maximum local SAR averaged over 10g of tissue is calculated by the software for a given input power. Additional simulations were conducted with the coil moved away from the skin, to assess how fast the absorption drops for increasing separation. The results are shown in Table 5 using an input power of 1W.

The simulations show that the maximum for each configuration is in the proximity of the Tx coil. Therefore, multiple simultaneously active coils are not expected to increase this value considerably. The indicated SAR values are the maximum values found in the tissue when the input power is 1W, corresponding to an input current of 0.74A. For a fixed current, the power will decrease when the coils are placed in the air. However, the exposure is related to the B-field, and hence to the current.

#### 3.4.2. Maximum Power Transfer

The values in Table 5 should be compared against the 2W/kg limit [59,60] for the general public. The maximum value of 4.3W/kg implies that the input power during continuous use should be maximally 0.46W when coils are placed close to the skin in the final design. When a belt increases the separation between the coil and skin surface to 5mm, the power that can be used increases to 0.68W. However, in a controlled environment (under the supervision of a professional), the SAR-limit for local exposure is 5 times higher, at 10W/kg. This implies that 3.4W input power can be used. For larger separations, the maximum possible input power is even higher. The efficiency, and therefore the power received, will be slightly lower as well due to the larger Tx–Rx distance, this effect will be smaller than the effect of the decreased exposure.

For an input power of 0.68W, the received power at the receiver is 18μW for the general public. The higher input power in a controlled environment can transfer 90μW to the receiver. If a higher power is needed, following the IEEE Guidelines [60], this will only be possible during a much shorter time interval: during each 100ms, 14mJ of power can be sent, taking into account the safety factor of 5 for non-continuous exposure as explained above. This means that 0.35μJ can be received every 100ms without restrictions on the duty cycle within this 100ms interval. This can be useful, e.g., for the start-up of a device.

### 3.5. PTE Comparison and Applications

#### 3.5.1. Comparison with Other Designs and Novelty

A comparison with other WPT designs for deep receivers is shown in Table 6. It can be seen that the combination of small receiver size and large WPT distance in tissue is what distinguishes the proposed design from existing systems.

For systems at the lower end of the frequency spectrum, [45,47,62], efficiency values are higher than the proposed design. While this can accommodate applications such as WCE, the dimensions of Tx and Rx are much larger. The radius of Rx is almost three times larger, while PTE typically increases with $\;r^{3}$ [44]. The larger receiver dimensions and still limited WPT distance (50–100 mm), make these designs unusable for the intended application of long-term monitoring for obese people. The objective of miniature receivers is not merely a niche application, it might be an actual requirement: bulky devices in the GI tract can hinder the passage of food [7], as opposed to WCE, where the capsule moves with the contents through the intestines. The large coils of the mentioned studies, worn around the body, also have disadvantages, as mentioned in Section 1.3.

The mid-field WPT design by Ho et al. [36], is a very relevant and regularly cited comparison, since these hlauthors were among the first to define and investigate mid-field powering ([49]), which is often considered optimal to power miniature, deep implants [32,46]. The received power of 195μW can be considered good for the small, 1 mm radius, receiver. However, it is mentioned in [36], that the power drops to 10μW for a WPT distance of 100 mm. At the maximum SAR level, this would be 22.5μW with an efficiency of $2\times 10^{-5}$. The presented work delivers a similar amount of power, 18μW, and higher efficiency at double the distance, 200 mm, although with a larger Rx radius. The decrease with a factor of 19.5 for a doubling of the WPT distance in [36], can indicate that the new, proposed design is more efficient at large depths compared to existing systems. A simulation of the proposed design indicated an improvement of a factor of 20.3 as well, for halving the WPT distance from 200 mm to 100 mm, which is larger than the estimated gain of the larger radius (×8).

Other designs in Table 6 propose radiative WPT, covering large distances of 500 mm and more. However, none of these designs transmit power through more than 60 mm of tissue. Therefore, interpreting these power and efficiency values for 200 mm deep receivers, would lead to an underestimation of losses and refraction in tissue, and consequently, overestimation of the efficiency.

This study is the first that provides efficiency and received power values for a WPT distance of a minimum of 200 mm in tissue and receivers with a maximum diameter of 4 mm. The corresponding opportunities and applications are discussed below.

**Table 6:** Comparison of the proposed WPT system with existing systems.

	Frequency (MHz)	Depth (mm)	Tx	Rx	PTE (%)	P_Tx_	P_Rx_	SAR (W/kg)
[45]	0.802	100	380 mm Ø (x2) ^a^	10 mm Ø	9.1	-	367 mW	-
[47]	1.0	100	200 mm Ø (x2)	8.9 mm Ø	1.0 ^b^	9.1 W	91 mW	0.66
[62]	13.56	50	45 mm Ø	11×11 mm^2^	0.03	0.3 W	-	0.04
[63]	655	500 ^c^	-	10 mm Ø	0.06	0.01 W	-	0.078
[64]	915	1500 ^c^	500 mm	10.8 mm Ø	0.0001	-	-	-
[36]	1600	50	60 × 60 mm^2^	2 mm Ø	0.04	-	195 µW	0.89
[36]	1600	100	60 × 60 mm^2^	2 mm Ø	0.002	-	10 µW	0.89
[65]	2340	200 ^c^	-	3×3 mm^2^	0.005 ^d^	-	100 µW	<0.1
[48]	2450	60	83×53×1.3 mm^3^	5×5.3 mm^2^	1.3	1 W	13 mW	0.90
This work	13.56	200	100 mm Ø × 10 mm	4 mm Ø	0.0027	0.7 W	19 µW	2.0

^a^ Two coils in a Helmholtz configuration. ^b^ With a ferrite core for Rx. ^c^ Only a small part of the transmission distance around Rx is tissue, the remaining part is air. ^d^ Efficiency calculated with optical power of the LED. Real value is higher.

#### 3.5.2. Applications

The value of 18μW for the received power is in line with the characteristics of long-term monitoring devices, as mentioned in Section 1.1. In [28], an ingestible thermometer is designed, with an energy consumption of 70μJ per measurement, and a stand-by power of 100 nW. A microbiome sensor by [15] consuming 12.7μW can now be used at depths up to 20 cm. A pressure sensor is designed by [66] consuming only 0.27μW, which can be used for GI tract motility sensing or bladder monitoring. A sensing and stimulation implant for the GI tract in [20] has sub-µW level power needs for motility recording and consumes power in the µW to mW range (depending on the stimulation parameters) during stimulation, which does not need to be continuous. The device is already wirelessly powered, although at a limited Tx–Rx separation. In general, advancements in ultra-low power miniature electronics enable sensing at increasingly lower power levels. Power levels can be <10 nW for ADCs and signal acquisition circuits, <nW stand-by power for sensor and processing nodes and <nW for timers [29,31]. A smart choice for the duty cycle for slightly more power-hungry components, e.g., stimulation or wireless communication, ensures the long-term functioning of the mentioned applications. The comparison of the obtained PTE and received power, with the power needs of modern hardware, shows that with the proposed system, sufficient power can now be transferred for long-term monitoring inside the abdominal cavity for virtually every patient. The large wavelength used eliminates focusing and the corresponding need to know the precise Rx position, and can accommodate charging multiple receivers.

## 4. Validation

As a validation of the simulation results, two realizations for both the Tx and Rx coil were manufactured and their performance was measured. The coils are connected to a vector network analyzer (VNA) operating at 13.56MHz from which the power transfer and Z-parameters are obtained indirectly from the S-parameters. The measurement results are shown in Table 7.

### 4.1. Transmitting Coil

**Figure 10:**
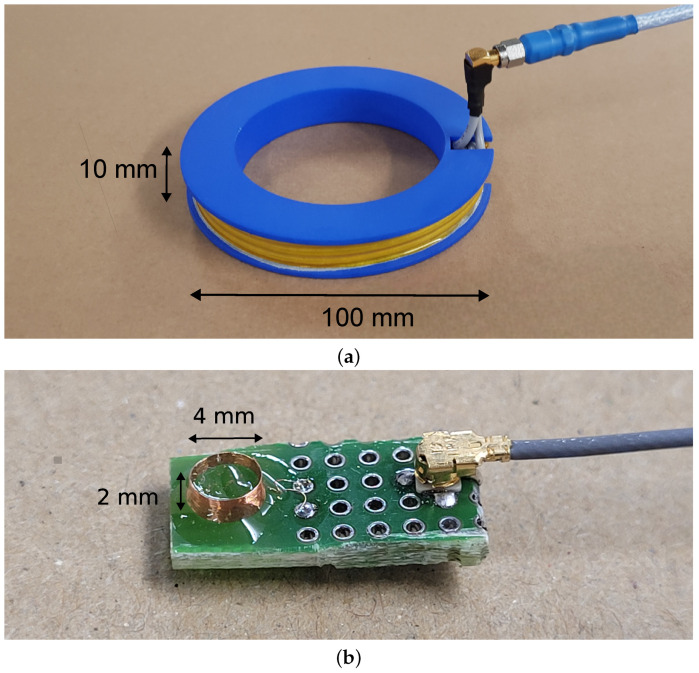
Prototypes of Transmitting and Receiving coils. (**a**) Prototype of the Transmitting coil with 12 windings of 1.3 mm in a 3 × 4 configuration. (**b**) Prototype of the Receiving coil with 23 windings of 0.08 mm in the axial direction.

The transmitting coil with 3 by 4 windings is put together according to the simulated specifications using a 3D-printed spool and commercial AWG 16 wire. The thickness of the insulation corresponds to half the winding spacing of 3mm. The Tx coil is shown in Figure 10a.

The inductance and capacitance of the coil are derived from the input impedance at different frequencies, and amount to 17.3μH and 12.4pF, respectively. While the inductance value matches the simulation, the capacitance is considerably larger. This can be attributed to the relative electric permittivity value of the insulation. The measured capacitance value was confirmed after taking into account the insulation in the simulation as well. However, the higher capacitance currently makes the coil behave capacitively at the frequency of 13.56 MHz, which will be addressed in future work.

### 4.2. Receiving Coil

The receiving coil, with 23 windings in the axial direction, is constructed on a small printed circuit board due to its miniature size. The coil is shown in Figure 10b.

The measured inductance and capacitance for the receiving coil are 2.26μH and 1.52pF, respectively. The same increase in capacitance can be seen for the transmitting coil, due to the presence of the polymer holding the coil together. Due to the smaller values for the inductance and capacitance, the coil is still in the inductive domain.

### 4.3. Power Transfer

Finally, the power transfer is measured for a distance of 200mm between Tx and Rx. The efficiency is calculated using the Z-parameters and Equation (2), which eliminates the need for a matched load, as well as a feeding circuit matched to the input impedance of Tx. The PTE 13.56MHz showed a lot of fluctuation, due to the small SNR. The average theoretical power transfer efficiency was $6\times 10^{-5}$, with up to a factor 2 of variations. This range corresponds with the simulated PTE value of $7.6\times 10^{-5}$ for power transfer in air. Since this is the theoretic efficiency value, using Equation (2), there is no influence of the higher capacitance mentioned in Section 4.1, which will need to be addressed to effectively obtain this PTE value. As opposed to mid-field and far-field wireless power transfer, the influence of the presence of tissue is limited to increased losses and therefore lower efficiency, as also indicated by the simulations. However, at this wavelength, tissue will not have an influence on the shape, beamforming or focusing of the field. Subsequently, measurements are limited to air at this point, and measurements with tissue will be part of future work investigating alignment and multiple transmitters as well.

## 5. Conclusions

The optimal frequency for wireless power transfer to a miniature, in-body receiver at depths up to 20 cm is investigated in the range of 1 kHz to 10 GHz. For the first time, this is explicitly examined for obese people, enabling WPT for virtually every body type. It is found that the optimal frequency coincides with the transition from dominant copper losses in the transmitter and receiver to dominant tissue losses due to induced currents. This point is close to the ISM frequency of 13.56 MHz, which was selected as the optimal value. As compared to more common higher frequency approaches, this frequency shows higher power transfer potential over distances of 20 cm in tissue to miniature receivers, without the need for focusing. This reduces requirements on implant position and focusing hardware, while also being more suited for WPT to multiple receivers.

A design for the transmitting and receiving coil is proposed, delivering optimal performance at 13.56 MHz, within the constrained space of 10 cm diameter for the transmitter and only 4 mm for the receiver. For the transmitter, a coil with 12 windings is designed, with an inductance of 17.3μH and a resistance of 3.7Ω next to the body. The optimal receiving coil has 18 windings, an inductance of 2.26μH and a resistance of 3.1Ω. Power transfer is simulated and validated by measurements. A PTE of $2.70\times 10^{-5}$ was found, corresponding to a received power of 18μW at the limit of maximum exposure. This study is the first to present efficiency and power values for WPT through 20 cm of tissue to miniature receivers. After comparison with the literature covering shallower depths or larger receivers, these values show the potential for a higher power budget and efficiency at large depths and will enable long-term monitoring with ingestible and implantable devices in the abdominal cavity. Applications can include pH, temperature and pressure measurements, motility sensing or even gastric stimulation.

Future work will cover the influence of misalignment on the PTE. Simultaneously, it will be investigated how multiple Tx coils can compensate for this while also increasing the received power, together with the corresponding measurements in air and tissue. An alternative design for the Tx coil with lower capacitance will be investigated in order to increase its self-resonance frequency.

## Figures and Tables

**Table 1:** Overview of the fixed geometric parameters and constraints.

Parameter	Value (mm)
Tx–Rx distance	200
Tx diameter	100
Tx thickness	10
Rx diameter	4
Rx thickness	2

**Table 2:** Overview of the tissue layers and thickness for the original and modified model.

Tissue Layer	Thickness (mm)	Thickness (mm)
	Original Model	Modified Model
Skin	2	2.5
Subcutaneous fat	42	56
Muscle	20	28
Visceral fat	124	96
Small intestine	12	17.5

**Table 3:** Design parameter ranges for transmitter and receiver.

Parameter		Minimum Value	Maximum Value
Tx	$N_{a}$	1	10
	$N_{r}$	1	10
	*d* (mm)	0.5	10
	$i_{a}$ (mm)	0.01	8
	$i_{r}$ (mm)	0.01	15
Rx	$N_{a}$	1	40
	$N_{r}$	1	4
	*d* (mm)	0.01	1.5
	$i_{a}$ (mm)	0.01	1.5
	$i_{r}$ (mm)	0.01	1.5

**Table 4:** Selection of 15 optimally performing Tx coil designs in terms of PTE vs. input resistance, defined by the number of windings in axial and radial direction, winding diameter and interwinding spacing in axial and radial direction.

$n_{a}$	$n_{r}$	*d* (mm)	$i_{a}$ (mm)	$i_{r}$ (mm)	$R_{1}$	PTE(−)
3	2	2.31	1.54	1.54	1.23	$1.33\times 10^{-5}$
3	3	1.67	2.50	2.50	2.41	1.32 $\times 10^{-5}$
3	4	1.30	3.04	3.04	3.66	$1.30\times 10^{-5}$
4	3	1.18	1.76	1.76	4.99	$1.28\times 10^{-5}$
4	4	1.18	1.76	1.76	7.95	$1.27\times 10^{-5}$
4	5	1.18	1.76	1.76	11.22	$1.24\times 10^{-5}$
5	4	1.11	1.11	1.11	14.29	$1.20\times 10^{-5}$
5	5	1.11	1.11	1.11	20.93	$1.16\times 10^{-5}$
5	6	1.11	1.11	1.11	28.30	$1.13\times 10^{-5}$
5	7	1.11	1.11	1.11	36.19	$1.09\times 10^{-5}$
5	8	1.11	1.11	1.11	44.47	$1.05\times 10^{-5}$
5	9	1.11	1.11	1.11	52.99	$1.01\times 10^{-5}$

**Table 5:** Exposure of the body during WPT for 10 configurations C1–C10 and an input power of 1 W.

	Max. 10 g SAR (W/kg) for 1 W Input Power		
	**Coil Next** **to Skin**	**Coil 1 mm** **away from Skin**	**Coil 5 mm** **away from Skin**
C1	4.3	4.0	2.9
C2	4.1	3.8	2.8
C3	4.2	3.9	3.0
C4	4.2	3.8	2.9
C5	4.3	3.9	2.9
C6	3.9	3.6	2.6
C7	3.8	3.4	2.5
C8	4.0	3.7	2.6
C9	2.8	2.5	1.9
C10	3.9	3.6	2.6
STD DEV	0.4	0.4	0.3
AVG	3.9	3.6	2.7

**Table 7:** Measured coil characteristics.

Quantity	Value
Tx Inductance L	17.3 μH
Tx Capacitance C	12.4 pF
Rx Inductance L	2.26 μH
Rx Capacitance C	1.52 pF

## Data Availability

The data presented in this study are available on request from the corresponding author.

## References

[1] Spaner S.J., Warnock G.L. (2009). A Brief History of Endoscopy, Laparoscopy, and Laparoscopic Surgery. J. Laparoendosc. Adv. Surg. Tech..

[2] Hopkins H.H., Kapany N.S. (1954). A flexible fibrescope, using static scanning. Nature.

[3] Iddan G., Meron G., Glukhovsky A., Swain P. (2000). Wireless capsule endoscopy. Nature.

[4] O’Grady J., Murphy C.L., Barry L., Shanahan F., Buckley M. (2020). Defining gastrointestinal transit time using video capsule endoscopy: A study of healthy subjects. Endosc. Int. Open.

[5] Maqbool S., Parkman H.P., Friedenberg F.K. (2009). Wireless capsule motility: Comparison of the smartPill® GI monitoring system with scintigraphy for measuring whole gut transit. Dig. Dis. Sci..

[6] Van Helleputte N., Even A.J., Leonardi F., Stanzione S., Song M., Garripoli C., Sijbers W., Liu Y.H., Van Hoof C. (2020). Miniaturized Electronic Circuit Design Challenges for Ingestible Devices. J. Microelectromech. Syst..

[7] Mau M.M., Sarker S., Terry B.S. (2021). Ingestible devices for long-term gastrointestinal residency: A review. Prog. Biomed. Eng..

[8] Liu X., Yang Y., Inda M.E., Lin S., Wu J., Kim Y., Chen X., Ma D., Lu T.K., Zhao X. (2021). Magnetic Living Hydrogels for Intestinal Localization, Retention, and Diagnosis. Adv. Funct. Mater..

[9] Palmer A. (1959). Basal body temperature determinations in the management of menstrual disorders. Clin. Obstet. Gynecol..

[10] Webster W.W., Smarr B. (2020). Using Circadian Rhythm Patterns of Continuous Core Body Temperature to Improve Fertility and Pregnancy Planning. J. Circadian Rhythm..

[11] Hasler W.L. (2014). The use of SmartPill for gastric monitoring. Expert Rev. Gastroenterol. Hepatol..

[12] Huang F., Magnin C., Brouqui P. (2020). Ingestible sensors correlate closely with peripheral temperature measurements in febrile patients. J. Infect..

[13] Tutuian R., Castell D.O. (2006). Review article: Complete gastro-oesophageal reflux monitoring – combined pH and impedance. Aliment Pharmacol Ther.

[14] Liao C.H., Cheng C.T., Chen C.C., Jow U.M., Chen C.H., Lai Y.L., Chen Y.C., Ho D.R. (2020). An Ingestible Electronics for Continuous and Real-Time Intraabdominal Pressure Monitoring. J. Pers. Med..

[15] Mimee M., Nadeau P., Hayward A., Carim S., Flanagan S., Jerger L., Collins J., Mcdonnell S., Swartwout R., Citorik R.J. (2018). An ingestible bacterial-electronic system to monitor gastrointestinal health. Science.

[16] Rezaei Nejad H., Oliveira B.C.M., Sadeqi A., Dehkharghani A., Kondova I., Langermans J.A.M., Guasto J.S., Tzipori S., Widmer G., Sonkusale S.R. (2019). Ingestible Osmotic Pill for In Vivo Sampling of Gut Microbiomes. Adv. Intell. Syst..

[17] Baltsavias S., Van Treuren W., Weber M.J., Charthad J., Baker S., Sonnenburg J.L., Arbabian A. (2020). In Vivo Wireless Sensors for Gut Microbiome Redox Monitoring. IEEE Trans. Biomed. Eng..

[18] De la Paz E., Maganti N.H., Trifonov A., Jeerapan I., Mahato K., Yin L., Sonsa-ard T., Ma N., Jung W., Burns R. (2022). A self-powered ingestible wireless biosensing system for real-time in situ monitoring of gastrointestinal tract metabolites. Nat. Commun..

[19] Sunwoo S.H., Lee J.S., Bae S.J., Shin Y.J., Kim C.S., Joo S.Y., Choi H.S., Suh M., Kim S.W., Choi Y.J. (2019). Chronic and acute stress monitoring by electrophysiological signals from adrenal gland. Proc. Natl. Acad. Sci. USA.

[20] Lo Y.K., Wang P.M., Dubrovsky G., Wu M.D., Chan M., Dunn J.C., Liu W. (2018). A Wireless Implant for Gastrointestinal Motility Disorders. Micromachines.

[21] Cigaina V. (2002). Gastric pacing as therapy for morbid obesity: Preliminary results. Obes. Surg..

[22] Xie W., Kothari V., Terry B.S. (2015). A bio-inspired attachment mechanism for long-term adhesion to the small intestine. Biomed. Microdevices.

[23] AUTOCAPSULE—Autonomous Multimodal Implantable Endoscopic Capsule for the Gastrointestinal Tract. https://www.autocapsule.eu/.

[24] Ghosh A., Li L., Xu L., Dash R.P., Gupta N., Lam J., Jin Q., Akshintala V., Pahapale G., Liu W. (2020). Gastrointestinal-resident, shape-changing microdevices extend drug release in vivo. Sci. Adv..

[25] Khan S.R., Pavuluri S.K., Cummins G., Desmulliez M.P. (2020). Wireless Power Transfer Techniques for Implantable Medical Devices: A Review. Sensors.

[26] Winters C., Subramanian V., Valdastri P. (2022). Robotic, self-propelled, self-steerable, and disposable colonoscopes: Reality or pipe dream? A state of the art review. World J. Gastroenterol..

[27] Rao K.M.P., Alotaibi F.M., Alkanfery H.M., Mehedi I.M., Rao K.P., Alotaibi F.M., Alkanfery H.M. (2023). Intelligent Wireless Capsule Endoscopy for the Diagnosis of Gastrointestinal Diseases. Diagnostics.

[28] Yoshida S., Miyaguchi H., Nakamura T. (2021). Development of Ingestible Thermometer with Built-in Coil Antenna Charged by Gastric Acid Battery and Demonstration of Long-Time in Vivo Telemetry. IEEE Access.

[29] Sheng H., Zhang X., Liang J., Shao M., Xie E., Yu C., Lan W., Sheng H., Zhang X., Liang J. (2021). Recent Advances of Energy Solutions for Implantable Bioelectronics. Adv. Healthc. Mater..

[30] Yoo S., Lee J., Joo H., Sunwoo S.H., Kim S., Kim D.H. (2021). Wireless Power Transfer and Telemetry for Implantable Bioelectronics. Adv. Healthc. Mater..

[31] Nadeau P., El-Damak D., Glettig D., Kong Y.L., Mo S., Cleveland C., Booth L., Roxhed N., Langer R., Chandrakasan A.P. (2017). Prolonged energy harvesting for ingestible devices. Nat. Biomed. Eng..

[32] Dinis H., Mendes P.M. (2021). A comprehensive review of powering methods used in state-of-the-art miniaturized implantable electronic devices. Biosens. Bioelectron..

[33] Pinton G., Aubry J.F., Bossy E., Muller M., Pernot M., Tanter M. (2012). Attenuation, scattering, and absorption of ultrasound in the skull bone. Med. Phys..

[34] Agarwal K., Jegadeesan R., Guo Y.X., Thakor N.V. (2017). Wireless Power Transfer Strategies for Implantable Bioelectronics. IEEE Rev. Biomed. Eng..

[35] Lecluyse C., Minnaert B., Kleemann M. (2021). A Review of the Current State of Technology of Capacitive Wireless Power Transfer. Energies.

[36] Ho J.S., Yeh A.J., Neofytou E., Kim S., Tanabe Y., Patlolla B., Beygui R.E., Poon A.S. (2014). Wireless power transfer to deep-tissue microimplants. Proc. Natl. Acad. Sci. USA.

[37] Huang Y., Yang P., Zhang Z. An ECG Acquisition System with Piezoelectric Energy Harvesting for Low Power Healthcare Devices. Proceedings of the International Conference on ASIC.

[38] Banks E., Lim L., Seubsman S.A., Bain C., Sleigh A. (2011). Relationship of obesity to physical activity, domestic activities, and sedentary behaviours: Cross-sectional findings from a national cohort of over 70,000 Thai adults. BMC Public Health.

[39] Wilmet G., Verlinde R., Vandevoorde J., Carnol L., Devroey D. (2017). Correlation between Body Mass Index and abdominal circumference in Belgian adults: A cross-sectional study. Rom. J. Intern. Med..

[40] Soares I.V., Gao M., Cil E., Sipus Z., Skrivervik A.K., Ho J.S., Nikolayev D. (2023). Wireless Powering Efficiency of Deep-Body Implantable Devices. IEEE Trans. Microw. Theory Tech..

[41] Iqbal A., Al-Hasan M., Mabrouk I.B., Basir A., Nedil M., Yoo H. (2021). Biotelemetry and Wireless Powering of Biomedical Implants Using a Rectifier Integrated Self-Diplexing Implantable Antenna. IEEE Trans. Microw. Theory Tech..

[42] Mahmood A.I., Gharghan S.K., Eldosoky M.A., Soliman A.M. (2023). Powering implanted sensors that monitor human activity using spider-web coil wireless power transfer. IET Power Electron..

[43] Yu Z., Chen J.C., He Y., Alrashdan F.T., Avants B.W., Singer A., Robinson J.T., Yang K. (2022). Magnetoelectric Bio-Implants Powered and Programmed by a Single Transmitter for Coordinated Multisite Stimulation. IEEE J. Solid-State Circuits.

[44] Singer A., Robinson J.T. (2021). Wireless Power Delivery Techniques for Miniature Implantable Bioelectronics. Adv. Healthc. Mater..

[45] Murliky L., Oliveira G., de Sousa F.R., Brusamarello V.J. (2022). Tracking and Dynamic Tuning of a Wireless Powered Endoscopic Capsule. Sensors.

[46] Campi T., Cruciani S., De Santis V., Maradei F., Feliziani M. (2019). Near field wireless powering of deep medical implants. Energies.

[47] Khan S.R., Desmulliez M.P. (2019). Towards a Miniaturized 3D Receiver WPT System for Capsule Endoscopy. Micromachines.

[48] Iqbal A., Sura P.R., Al-Hasan M., Mabrouk I.B., Denidni T.A. (2022). Wireless power transfer system for deep-implanted biomedical devices. Sci. Rep..

[49] Poon A.S., O’driscoll S., Meng T.H. (2010). Optimal frequency for wireless power transmission into dispersive tissue. IEEE Trans. Antennas Propag..

[50] Kim S., Ho J.S., Poon A.S. (2012). Wireless power transfer to miniature implants: Transmitter optimization. IEEE Trans. Antennas Propag..

[51] Freeman D.K., Byrnes S.J. (2019). Optimal Frequency for Wireless Power Transmission into the Body: Efficiency Versus Received Power. IEEE Trans. Antennas Propag..

[52] Soares I.V., Gao M., Skrivervik A.K., Sipus Z., Zhadobov M., Sauleau R., Nikolayev D. Physical bounds on implant powering efficiency using body-conformal WPT systems. Proceedings of the 2021 IEEE Wireless Power Transfer Conference, WPTC.

[53] Gosselin M.C., Neufeld E., Moser H., Huber E., Farcito S., Gerber L., Jedensjo M., Hilber I., Gennaro F.D., Lloyd B. (2014). Development of a new generation of high-resolution anatomical models for medical device evaluation: The Virtual Population 3.0. Phys. Med. Biol..

[54] COMSOL Multiphysics v5.6. COMSOL AB, Stockholm, Sweden. https://www.comsol.com.

[55] Gabriel C. (1996). Compilation of the Dielectric Properties of Body Tissues at RF and Microwave Frequencies.

[56] Jayathurathnage P., Vilathgamuwa M., Simovski C. Revisiting Two-Port Network Analysis for Wireless Power Transfer (WPT) Systems. Proceedings of the 2018 IEEE 4th Southern Power Electronics Conference, SPEC 2018.

[57] Jiles D.C. (1994). Modelling the Effects of Eddy Current Losses on Frequency Dependent Hysteresis in Electrically Conducting Media. IEEE Trans. Magn..

[58] (2002). Standard Specification for Standard Nominal Diameters and Cross Sectional Areas of AWG Sizes of Solid Round Wires Used as Electrical Conductors.

[59] International Commission on Non-Ionizing Radiation Protection (2010). Guidelines for limiting exposure to time-varying electric and magnetic fields (1 Hz–100 kHz). Health Phys..

[60] IEEE (2019). C95.1-2019—IEEE Standard for Safety Levels with Respect to Human Exposure to Electric, Magnetic, and Electromagnetic Fields, 0 Hz to 300 GHz.

[61] Zurich MedTech (2018). Sim4Life v6.2.

[62] Lyu H., John M., Burkland D., Greet B., Post A., Babakhani A., Razavi M. (2020). Synchronized Biventricular Heart Pacing in a Closed-chest Porcine Model based on Wirelessly Powered Leadless Pacemakers. Sci. Rep..

[63] Abdi A., Aliakbarian H. (2019). A Miniaturized UHF-Band Rectenna for Power Transmission to Deep-Body Implantable Devices. IEEE J. Transl. Eng. Health Med..

[64] Ding S., Koulouridis S., Pichon L. (2020). Implantable wireless transmission rectenna system for biomedical wireless applications. IEEE Access.

[65] Park S.I., Brenner D.S., Shin G., Morgan C.D., Copits B.A., Chung H.U., Pullen M.Y., Noh K.N., Davidson S., Oh S.J. (2015). Soft, stretchable, fully implantable miniaturized optoelectronic systems for wireless optogenetics. Nat. Biotechnol..

[66] Danneels H., Coddens K., Gielen G. A fully-digital, 0.3V, 270 nW capacitive sensor interface without external references. Proceedings of the European Solid-State Circuits Conference.

